# Using PCR-Based Sequencing to Diagnose *Haycocknema perplexum* Infection in Human Myositis Case, Australia

**DOI:** 10.3201/eid2412.181240

**Published:** 2018-12

**Authors:** Anson V. Koehler, Peter Leung, Belinda McEwan, Robin B. Gasser

**Affiliations:** University of Melbourne, Parkville, Victoria, Australia (A.V. Koehler, R.B. Gasser);; Royal Hobart Hospital, Hobart, Tasmania, Australia (P. Leung, B. McEwan)

**Keywords:** myositis, humans, nematodes, Australia, Tasmania, Haycocknema perplexum, PCR, parasites

## Abstract

We report a case of myositis in a male patient in Australia who had progressive weakness and wasting in his left lower limb. Although clinical, pathologic, and laboratory assessments were inconclusive, a new, nested PCR–coupled sequencing method enabled the unequivocal diagnosis of myositis caused by the enigmatic nematode *Haycocknema perplexum*.

In 2017, a 37-year-old male resident of the eastern coast of Tasmania, Australia, arrived at Royal Hobart Hospital reporting a 2-year history of progressive muscle weakness and wasting in his left lower limb. He had also developed weakness in his left upper limb 6 months before clinical examination. Initially, he underwent a neurologic assessment for motor neurone disease before being referred to the hospital. The patient is a recreational hunter and consumes bushmeat; his only travel history was to Melbourne, Victoria, Australia.

Clinical examination revealed that this patient walked with a mild limp and a high stepping gait on the left. The Romberg test result was unremarkable, and tandem gait and coordination were normal. There was marked wasting of the left vastus lateralis and the gastrocnemius muscles and mild wasting of the left biceps and triceps muscles. There were no associated fasciculations. Muscle power was reduced on the left side, with weakness of hip flexion (3+/5), knee extension (4+/5), and flexion (3+/5). The patient was unable to squat fully. Reflexes were reduced on the left compared with the right side, and plantar reflexes were recorded as normal bilaterally. No objective sensory deficit was detected. Cranial nerve function, specifically extraocular muscles, speech, and tongue power and movement, appeared normal. Axial muscles appeared to function normally.

Complementary serologic tests revealed no evidence of antibodies for *Toxocara*, *Toxoplasma*, *Trichinella*, or *Strongyloides*. Test results for serum calcium, magnesium, and phosphate were within reference ranges, but serum creatinine kinase was elevated at 3,636 IU/L (reference range 22–198). Electrophoretic analysis for multiple myeloma revealed a diffuse increase in gamma globulin but no paraprotein. Liver function tests revealed that levels of alanine aminotransferase (139 IU/L) and aspartate aminotransferase (94 IU/L) were elevated, whereas urea electrolytes and creatinine were within reference ranges. Full blood examination revealed a transient mild eosinophilia (0.54 × 10^9^ cells/L).

Magnetic resonance imaging did not detect abnormalities in the brain or spinal cord, although a posterior disk protrusion relating to a slight central canal stenosis was observed. Nerve conduction results were within reference range, but electromyographic evaluations revealed active and chronic denervation changes confined to the left upper and lower limbs, consistent with a myopathic process. Histopathologic examination of a biopsy specimen (20 × 20 × 5 mm^2^) taken from the left vastus lateralis muscle revealed a chronic, destructive myopathy with inflammation, including eosinophils. This examination detected variation in muscle fiber size and patchy foci of inflammatory cell infiltrates, predominantly lymphocytic, but also with scattered eosinophils and some active myocyte destruction. There was an increase in interstitial fibrosis; many of the fibers appeared degenerative, some showed extensive vacuolation, and others were in the process of regenerating. Fibers appeared split, and some of them had internal nuclei. Occasional fibers had inclusions within the sarcoplasm, which appeared rounded, with central irregular eosinophilic and brown areas. No glycogen or lipid was found within vacuoles, and there was evidence of some cytochrome oxidase depletion. The muscle appeared to be type I dominant with atrophic type 1 and 2 fibers. 

On the basis of these findings, we made a diagnosis of chronic destructive myopathy with inflammation (including eosinophils), with differential diagnoses of inclusion body myositis and parasitic myositis. We conducted examinations for autoimmune disorders, but results for dsDNA, antinuclear antibody, and extractable nuclear antigen tests were within reference ranges.

When the patient’s condition showed no clinical improvement, we administered therapeutic doses of albendazole (400 mg 2×/d) for 3 months. The patient’s clinical status improved, and creatinine kinase levels in serum decreased to 387 IU/L. He remained on surveillance as an outpatient for 7 months. On the basis of this clinical evidence and the patient’s history, we suspected an infection with *H. perplexum* nematodes.

From a 2-mm^3^ portion of the same muscle biopsy sample used for the previous histopathologic examination and frozen, we extracted genomic DNA using the DNeasy PowerSoil Kit (QIAGEN, Venlo, the Netherlands) and then subjected 2-μL aliquots of this DNA sample to 2 new nested PCR assays adapted from an established method (*1**–**3*) that had not amplified products from the same sample. The 2 optimized nested PCRs (Table 1) successfully amplified products of the expected sizes (≈400 bp for *cox-1* and ≈830 bp for *SSU*) from this sample, and these amplicons were directly sequenced (*3*).

**Table 1:** Primer sequences for nested PCR to detect *Haycocknema perplexum* nematodes using partial regions of the *cox*-1 gene and the *SSU* gene*

Designation	Primer pair	Oligonucleotide sequence, 5′ → 3′	Annealing temperature, °C	Expected size, bp	Reference
*cox*-1					
1° PCR	RhigoCoxF	TTTTTTGGACATCCTGAGGTGTAT			(*3*)
	RhigoCoxR	CAGACTCAACACATAATGAAAATG	47	450	(*3*)
2° PCR	HPCO1F	GGACATCCTGAGGTGTATAT			This study
	HPCO1R	AATGAAAATGTCCTACCACA	50	400	This study
*SSU*					
1° PCR	Nem18sF	CGCGAATRGCTCATTACAACAGC			(*1*)
	1912R	TTTACGGTCAGAACTAGGG	54	900	(*2*)
2° PCR	1096F	GGTAATTCTGGAGCTAATAC			(*2*)
	Nem18sR	GGGCGGTATCTGATCGCC	54	830	(*1*)

*All PCR used 35 cycles with initial extension of 5 min. All cycles were 30 s except that *SSU* had a 1 min extension for PCRs. *cox*-1, cytochrome *c* oxidase 1; *SSU*, small subunit of nuclear ribosomal RNA.

We assessed the sequences obtained (GenBank accession nos. MH667568 [*cox-*1] and MH667568 [*SSU*]) for quality, compared them (using blastn, https://www.ncbi.nlm.nih.gov/BLAST) with publicly available sequences in the GenBank database to confirm the diagnosis of *H. perplexum* nematode infection, and then aligned them with respective reference sequences from this database, as described previously (*3*), to assess genetic variability. From the resultant *cox*-1 amplicon, we obtained 385 bp of clean sequence that varied by 1.3%–5.7% from previously determined *cox*-1 sequences from *Haycocknema* nematodes (GenBank accession nos. KU531719 and KU531720, representing Tasmania and Queensland, respectively [*3*]). As expected, the *cox*-1 sequences from samples from Tasmania were more closely related to one another than to 1 from Queensland. A comparison of all three *cox*-1 sequences of *H. perplexum* nematodes (>348 nt) revealed 1 nonsynonymous and 24 synonymous mutations. From the resultant *SSU* amplicon, we obtained 831 bp of clean sequence, which varied by 0.5%–0.6% from *SSU* sequences reported previously for *Haycocknema* nematodes (GenBank accession nos. KU531721 and KU531722 representing Tasmania and Queensland, respectively [*3*]), whereas the sequences KU531721 and KU531722 differed by only 0.1% (1 nt).

## Conclusions

This case was consistent clinically with some previous cases (*3**–**9*) associated with *H. perplexum* infection in Tasmania and Queensland (Table 2). PCR-coupled sequencing enabled an etiologic diagnosis, although this diagnosis was not possible based on clinical, clinicopathologic, and histopathologic findings. The analytical sensitivity of the nested PCRs allowed for the ready and specific detection of *H. perplexum* DNA within the muscle DNA sample. The genetic distinctiveness recorded here was also seen previously between samples from cases from Tasmania and Queensland. Although it is speculative, this information lends additional support for some population genetic substructuring within *Haycocknema* (because of geographic or reproductive isolation). It is possible that the 3 distinct genotypes recorded may differ in their biology, host affiliation, transmission pattern(s), or a combination of these factors if they turn out to be transmitted from animals to humans.

**Table 2:** Recorded human cases of *Haycocknema perplexum* infection in Australia (*8**,**9*)*

Patient no., age, y/sex	Year of diagnosis	State†	Travel	Animal contact	Symptom duration, y	Maximum CK, U/L‡	Eosinophils, × 10^9^ cells/L	Reference
1, 33/F	1994	TAS	Global, NT	Botanist, fieldwork, vegetarian, some bushmeat consumption	5	3,294	0.8	(*4,6,10*)
2, 48/M	1996	TAS	Far north QLD, NT	NA	1.5	1,586	2.0	(*4–6*)
3, 61/M	2004	QLD	None in 20 years, raised in TAS	NA	3	1,263	High	(*6,7*)
4, 23/F	2005	QLD	WA, NSW, VIC	NA	2	1,370	1.1	(*7*)
5, 61/M	2006	QLD	Regional	Never consumed bushmeat	2	1,230	1.36	(*7*)
6, 50/M	2011	TAS	Born Tanzania; Ireland, NSW	Extensive contact with native animals	2	5,700	Normal	(*8*)
7, 72/M	2015	TAS	NA	Recreational hunter	>2	2,082	2.44	(*3*)
8, 30/M	2015	QLD	Regional	Never consumed bushmeat	2	3,400	1.24	(*3*)
9, 80/F	2012	QLD	Extensive TAS	Native animal carer	1.5	270	0.7	(*9*)
10, 37/M	2016	TAS	VIC	Recreational hunter	2	3,636	0.54	This study

*CK, creatinine kinase; NA, not available; NSW, New South Wales; NT, Northern Territory; QLD, Queensland; TAS, Tasmania; VIC, Victoria; WA, Western Australia.  †All patients from QLD were from towns in far north QLD. ‡Reference range <170. §Reference range <0.4.

There has been considerable controversy surrounding the biology and transmission of *H. perplexum* nematodes (*3**,**5*). It appears that only 4 of 9 cases of human infection recorded to date (the case we report and 3 previous cases [*7**,**8**,**10*]) had histories of the patients consuming bushmeat, which suggests the possibility of other routes of transmission. Other than locations in Tasmania and northern Queensland, the common thread among most clinical cases is an association with an exposure to native wildlife (e.g., through hunting, bush walking, caring for wildlife, keeping wildlife as pets, doing botanical fieldwork, or consuming native bushmeat [*3**,**4**,**6**–**9*]). However, Koehler et al. (*3*) suggested that *H. perplexum* nematodes might use an arthropod for transmission to humans. Clearly, the mysteries surrounding *H. perplexum* nematodes, including taxonomic classification, biology, reservoir host animals, and transmission to humans to cause disease, provide exciting paths for future research. The nested PCR–based sequencing approach we used will aid in such research.
